# Chest X-ray findings in late-onset congenital diaphragmatic hernia, a rare emergency easily misdiagnosed as hydropneumothorax: a case report

**DOI:** 10.1186/s13256-015-0755-9

**Published:** 2015-12-22

**Authors:** Vassil Nikolov Zefov, Maryam Anas Almatrooshi

**Affiliations:** Radiology Department, Dubai Health Authority, Latifa Hospital, Oud Metha Road, Dubai, United Arab Emirates

**Keywords:** Congenital diaphragmatic hernia, Hydropneumothorax, Chest pain, Abdominal gas pattern

## Abstract

**Introduction:**

Late-onset congenital diaphragmatic hernia is a rare anomaly with misleading symptoms and signs.

**Case presentation:**

We describe the case of a 7-year-old Middle Eastern girl who presented with acute nonspecific abdominal symptoms and respiratory distress of 2 days’ duration after sustaining a blunt trauma on her left chest wall on a background of chronic ill-defined left chest pain of 2 weeks’ duration. Her initial chest radiograph showed an air-fluid level, which was thought to be a hydropneumothorax, so a chest tube was inserted and was shown to be positioned between the chest wall and the air collection; therefore, a nasogastric tube was inserted and it was positioned in the left chest cavity so the diagnosis of late-onset congenital diaphragmatic hernia was made. On retrospective analysis of the first abdominal X-ray, it showed a subtle lucent area that was triangular in shape and continued with the chest cavity, which indicates a sign of diaphragmatic hernia. In addition, the next unusual point was the nonvisualization of the diaphragm, which should be reported in any abdominal X-ray. An exploratory laparotomy was performed on our patient using a left-sided subcostal incision; the operative findings revealed a very small posterior rim of the diaphragm and a hypoplastic left lung. Her stomach, spleen, and left colon with the omentum were in the left side of her chest. She made an uneventful recovery postoperatively and was discharged after 1 week.

**Conclusions:**

Gastric and intestinal gas shadow distribution provides an important marker in the diagnosis of late-onset congenital diaphragmatic hernia and should be sought for in every case of suspected congenital diaphragmatic hernia in addition to noting the position of the nasogastric tube in the chest cavity.

## Introduction

Congenital diaphragmatic hernia (CDH) is a life-threatening anomaly that usually presents antenatally or soon after birth [1, 2]. Its exact pathogenesis is not well understood [1]. Most newborn infants present with severe respiratory compromise in the neonatal period [3]. Late presentation is rare and variable leading to misleading radiologic assessment, delay in treatment and fatal consequences [3, 4]. Radiologically, CDH can mimic congenital lung cysts, infective lung diseases and pneumothoraces [4]. We report the case of a patient with a rare presentation of late-onset CDH resembling hydropneumothorax and review the literature.

## Case presentation

**Fig. 1:**
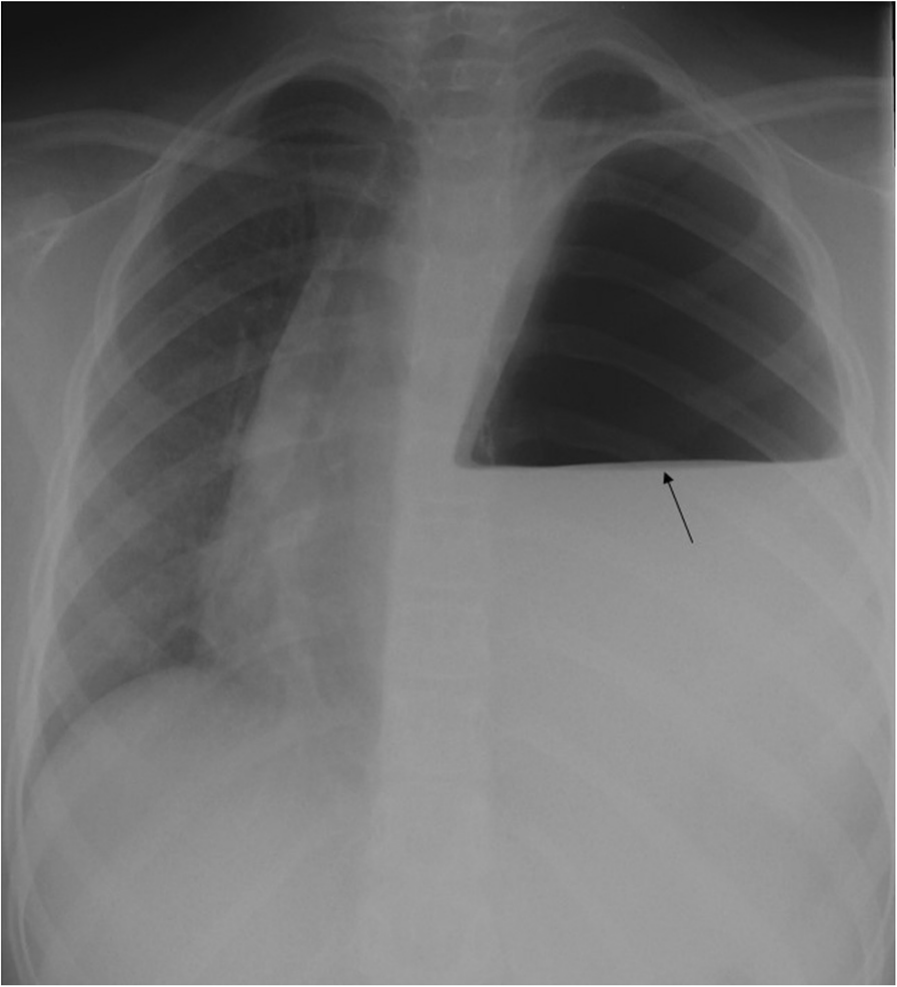
**Arrow showing air fluid level occupying the entire left chest**

**Fig. 2:**
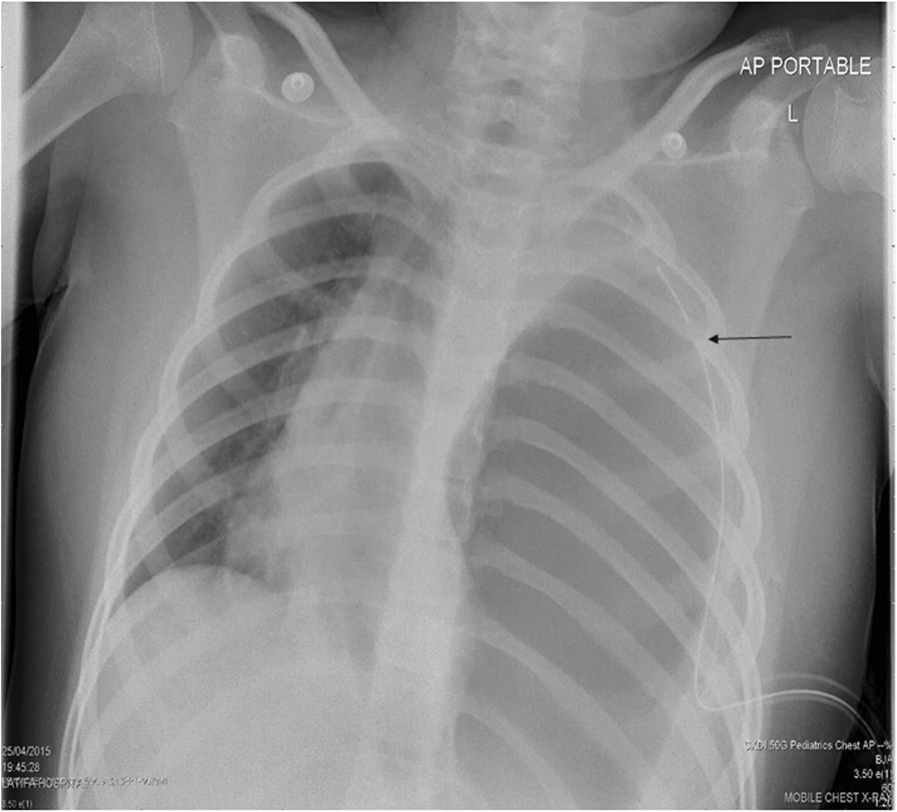
**Arrow showing tube positioned between the chest wall and the air collection**

**Fig. 3:**
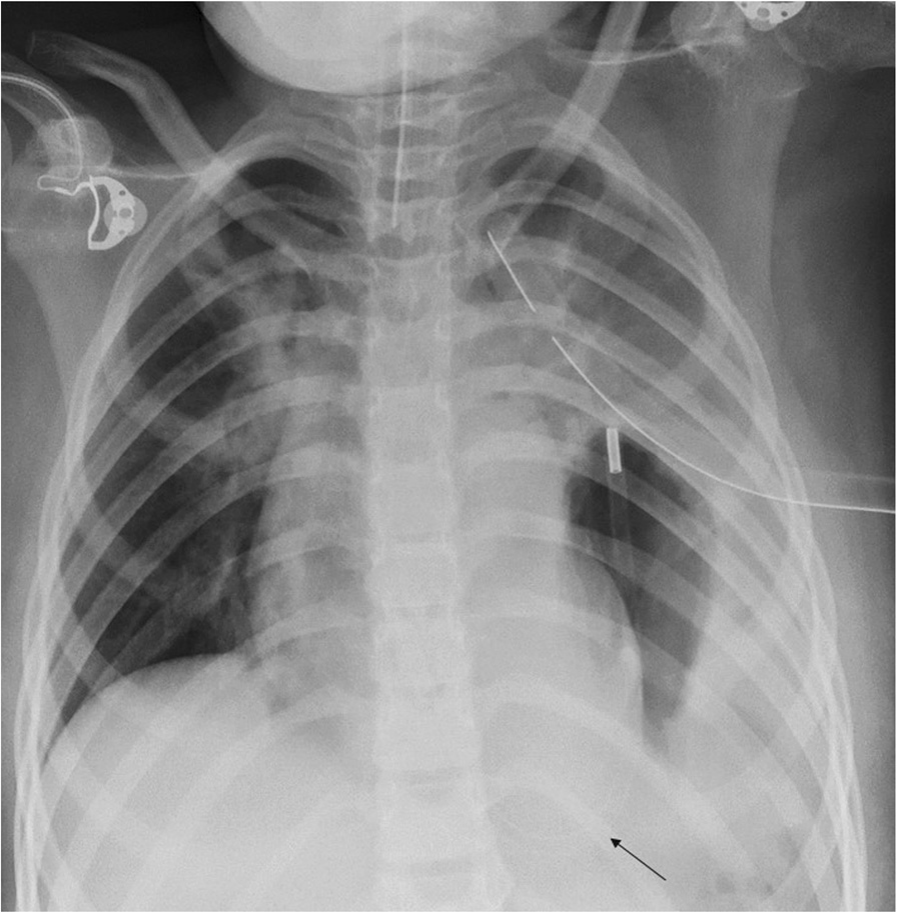
**Arrow showing the tip of the nasogastric (NG) tube positioned in the left side of the chest**

**Fig. 4:**
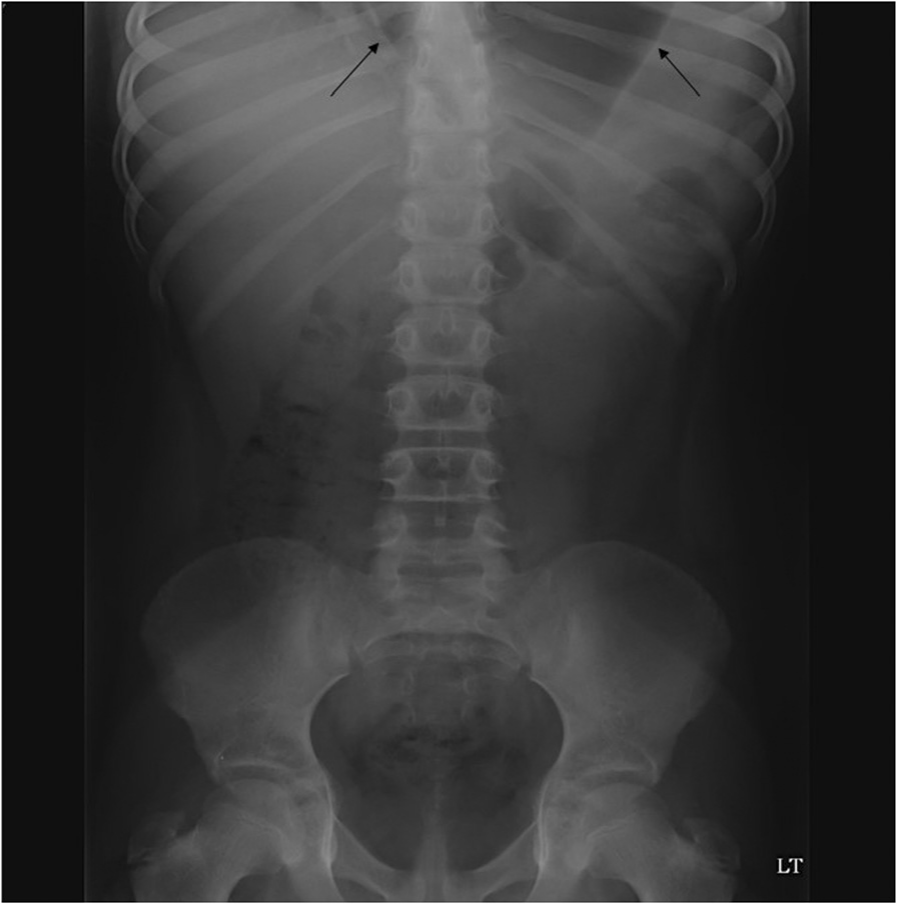
**Arrow showing a triangular lucent area in the upper abdomen connected to the left chest cavity**

A 7-year-old Middle Eastern girl presented with acute vomiting, upper abdominal pain of 2 days’ duration, which started after she fell out of bed while sleeping, and left-sided chest pain of 2 weeks’ duration, radiating to her left shoulder, aggravated by deep breathing and relieved with sleeping on two pillows at night; she had even gradually lost weight over the last few weeks. Two of her siblings had had pneumonia during this month. On physical examination, she appeared in respiratory distress, tachypneic, maintaining an oxygen saturation of 90 % on room air with markedly reduced air entry and chest movements over the left side. A small bruise was noted on the left side of her chest. Her abdomen was soft to palpation with no peritoneal signs and no epigastric mass. An initial chest radiograph (Fig. 1) showed an air-fluid level occupying her entire left chest so a chest tube was inserted on suspicion of hydropneumothorax. A chest radiograph following the chest tube insertion (Fig. 2) showed the tube positioned between the chest wall and the air collection and not draining any fluid, which raised the suspicion of congenital diaphragmatic hernia. After nasogastric (NG) tube insertion, the chest radiograph (Fig. 3) showed the tip of the NG tube positioned in the left side of her chest. A retrospective analysis of the abdominal X-ray (Fig. 4) showed a triangular lucent area in her upper abdomen connected to her left chest cavity indicating a missed diaphragmatic hernia.

**Fig. 5:**
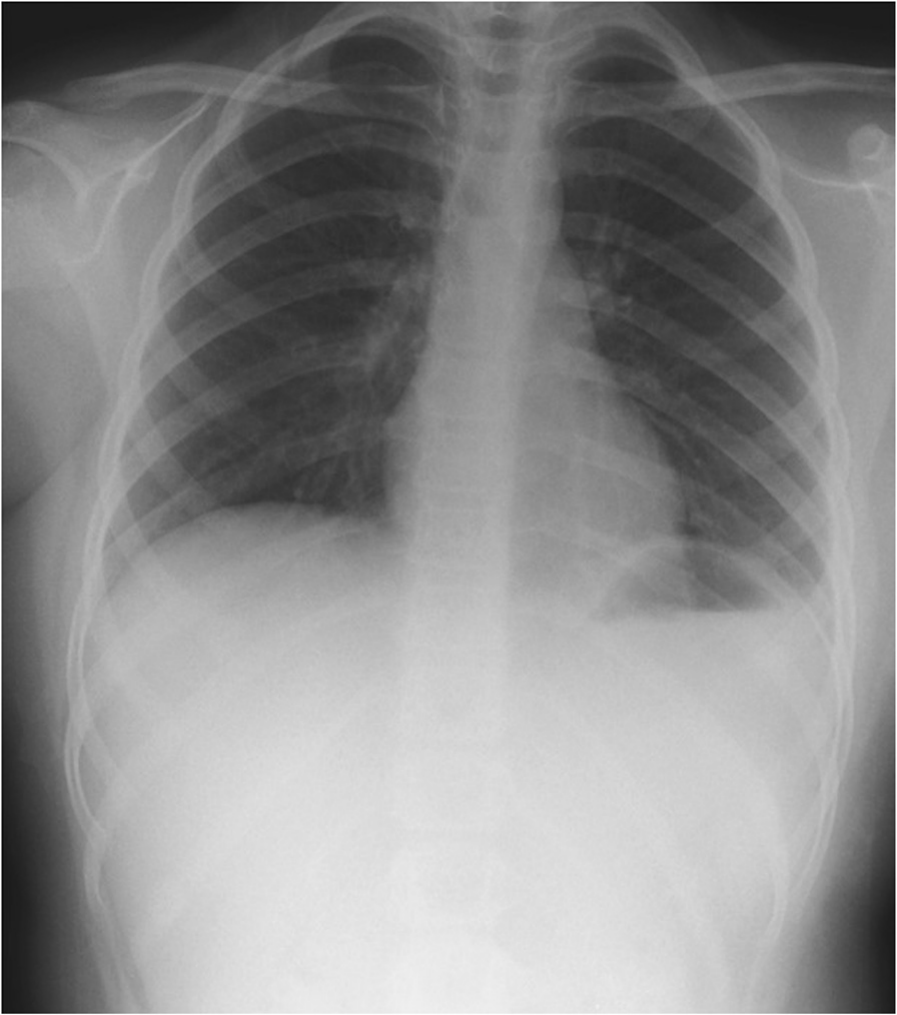
**Showing normal appearance of the left diaphragm with normal lung expansion and stomach shadow below diaphragm**

Our patient was admitted to the intensive care unit (ICU), intubated, ventilated and nasogastric fluid was aspirated, she was then transferred under pediatric surgery care to the operating theater. Our patient underwent an exploratory laparotomy with a left-sided subcostal incision; the operative findings revealed a very small posterior rim of the diaphragm and a hypoplastic left lung. Her stomach, spleen, and left colon with the omentum were in the left side of her chest. Her stomach and, left colon and the omentum were reduced easily but there was some difficulty reducing her spleen back into her abdomen. Our patient tolerated the entire procedure fairly well. The postoperative chest radiograph (Fig. 5) showed the normal appearance of the left diaphragm with normal lung expansion and the stomach shadow below the diaphragm. She made an uneventful recovery postoperatively and was discharged after 1 week. Her follow-up visits were unremarkable.

## Discussion

The diaphragm is formed between the eighth and tenth week of gestational age by fusion of the pleuroperitoneal membrane with the transverse septum thereby dividing the coelomic cavity into the abdominal and thoracic cavity, so the process of herniation of the abdominal content into the thoracic cavity occurs before completion of the closure of the pleuroperitoneal canal [3]. Congenital diaphragmatic hernia (CDH) occur in newborn infants in approximately 1 in 2,500 live newborns [5], correct prenatal diagnosis occurs in 50–90 % percent of the cases with antenatal ultrasonography [6] and only 5 % of diaphragmatic hernia occur beyond the neonatal period [4]. In the literature, presentation of late-onset diaphragmatic hernia can range from frequent respiratory tract infections, wheeze and mild gastroenteritis to cardiorespiratory failure [3–5], similar to our case, where our patient presented with nonspecific gastrointestinal symptoms and respiratory distress that rapidly progressed to respiratory failure and intubation, which shows the importance of early diagnosis and detection. Furthermore, the bruise on the left side of her chest added to the confusion.

Because most diaphragmatic hernias occur on the left side of the chest, most frequently Bochdalek hernia, a large number of them allow the stomach to enter into the chest cavity [5]. Although the diaphragmatic defect is present in fetal life, the occurrence of postnatal diaphragmatic hernia has been reported after months or years of normal chest radiographs, probably due to the effect of liver protection on the right side and spleen protection on the left side and the presence of a thin hernia sac, which contains both pleura and peritoneum [5]. Increase in abdominal pressure can precipitate the protrusion of the abdominal content into the thoracic cavity [4, 5], which we believe happened to our patient, as the previous history of her fall out of bed caused a significant increase in abdominal cavity pressure and the blunt trauma to her chest wall aggravated the preexisting left-sided congenital diaphragmatic hernia.

It is well known that pneumothorax is a life-threatening complication that occurs in 30–39 % of patients after a chest trauma, and failure to treat it can result in patient death [7]. The main diagnostic modality for detection of pneumothorax or hydropneumothorax is plain radiography and computed tomography [8]. The radiologic signs that establish the diagnosis of hydropneumothorax include the horizontal line of an air-fluid level, fluid effusion and collapse of the lung [8], which explain the diagnostic confusion and overlap that occurred with the initial chest radiography (Fig. 1), thus a chest tube was inserted to drain the hydropneumothorax. The suspicion of diaphragmatic hernia was raised on the appearance of the tip of chest tube between the chest wall and air collection (Fig. 2). The inserted NG tube showed its tip in her left chest cavity so the diagnosis was confirmed. The abdominal X-ray (Fig. 4) showed a triangular lucent area in her left upper abdomen, which we believe was an important marker that was missed and reported as normal. Careful attention should be paid to intestinal gas shadow patterns, especially in the upper abdomen, before reporting them as normal.

Most patients with late-onset congenital diaphragmatic hernia do well after surgical repair with apparently normal ipsilateral lung volume [5], which was expected in our patient post surgery.

## Conclusions

Late-onset congenital diaphragmatic hernia is a tricky diagnosis with misleading symptoms and signs with a wide range of differential diagnoses, so careful examination of any initial abdominal X-ray that shows a sign of gastric and intestinal gas shadow connection with the chest cavity will help dramatically in the diagnosis of suspected diaphragmatic hernia. The position of the nasogastric tube in the chest cavity will provide an important indicator and prompt correct diagnosis.

## Consent

Written informed consent was obtained from the patient’s guardian for publication of this case report and any accompanying images. A copy of the written consent is available for review by the Editor-in-Chief of this journal.

## Abbreviations

CDH: congenital diaphragmatic hernia
ICU: intensive care unit
NG: nasogastric
